# Supplementary material for: Multiple conserved cell adhesion protein interactions mediate neural wiring of a sensory circuit in C. elegans
Source: eLife. 2017 Sep 13;6:e29257. doi: 10.7554/eLife.29257 (PMC5619950; doi:10.7554/eLife.29257)
Supplement: Supplementary file 2. [file elife-29257-supp2.docx]

**Supplementary File 2. *C. elegans* strains, molecular cloning and transgenes**

**Fluorescent reporter strains for cell adhesion gene expression**

| Strain name | Transgene | Strain background | Reporter for |
| --- | --- | --- | --- |
| EM1162 | *sEx10331[rCesB0034.3a::GFP + pCeh361]*^1^ | *dpy-5(e907) I; him-5(e1490) V* | *casy-1* |
| EM1176 | *sEx12431[rCesC09E7.3::GFP + pCeh361]*^1^ | *dpy-5(e907) I; him-5(e1490) V* | *oig-1* |
| EM1191 | *sIs10411[rCes B0457.1::GFP + pCeh361]*^1^ | *dpy-5(e907) I; him-5(e1490) V* | *lat-1* |
| EM1168 | *sIs13247[rCesC40C9.5::GFP + pCeh361]*^1^ | *dpy-5(e907) I; him-5(e1490) V* | *nlg-1* |
| EM1195 | *hdEx333[rig-6a::YFP + pha-1(+)]*^2^ | *pha-1(e2123) III; him-5(e1490) V* | *rig-6* |
| EM1199 | *hdEx335[ncam-1a::YFP + pha-1(+)]*^2^ | *pha-1(e2123) III; him-5(e1490) V* | *ncam-1* |
| EM1200 | *hdEx341[igcm-1::YFP + pha-1(+)]*^2^ | *pha-1(e2123) III; him-5(e1490) V* | *igcm-1* |
| EM1188 | *otIs25[zig-1::GFP + pRF4(rol-6)]*^3^ | *him-5(e1490) V* | *zig-1* |
| EM1205 | *bxEx170[oig-8p::GFP + pRF4(rol-6)]*^4^ | *him-5(e1490) V* | *oig-8* |
| EM1278 | *bxEx228[sax-3p::YFP + pRF4(rol-6)]*^4^ | *him-5(e1490) V* | *sax-3* |
| EM1254 | *bxEx204[bam-2p::YFP + pCeh361]*^4^ | *dpy-5(e907) I; him-5(e1490) V* | *bam-2* |
| EM1282 | *bxEx232[wrk-1p::YFP + pCeh361]*^4^ | *dpy-5(e907) I; him-5(e1490) V* | *wrk-1* |
| EM1294 | *bxEx244[sax-7p::YFP + pCeh361]*^4^ | *dpy-5(e907) I; him-5(e1490) V* | *sax-7* |

^1^Original transgene was previously described (Hunt-Newbury et al., 2007).

^2^Original transgene was previously described (Schwarz et al., 2009).

^3^Original transgene was previously described (Aurelio et al., 2002).

^4^Transgene generated in this study

**Molecular cloning and constructs**

*eat-4pA::gfp*, *eat-4pA::wCherry*, and *eat-4pA::mCherry::RAB-3*

*eat-4pA*, a 419 bp promoter fragment of the *eat-4* gene (-5457 ~ -5039 bp), was PCR-amplified from *pPC52* (a gift from L. García) with restriction sites of SphI and XmaI. This PCR product was digested and ligated into SphI/XmaI-digested *pPD95.75* vector to generate *eat-4pA::gfp*, into SphI/XmaI-digested *inx-18p::wCherry* (Oren-Suissa et al., 2016) to generate *eat-4pA::wCherry*, or into SphI/XmaI-digested *pkd-2p::mCherry::RAB-3* (a gift from M. Lázaro-Peña) to generate *eat-4pA::mCherry::RAB-3*. *eat-4pA* drives expression in the male-specific sensory neurons HOA and PCAs.

*inx-18p::gfp*, *inx-18p::wCherry,* and *inx-18p::TagBFP*

*inx-18p::wCherry* was described previously (Oren-Suissa et al., 2016). To generate *inx-18p::gfp*, *inx-18p* fragment was digested with SphI and XmaI from *inx-18p::wCherry* and cloned into SphI/XmaI-digested *pPD95.75* vector. *inx-18p::TagBFP* was obtained by a PCR-fusion method (Hobert, 2002). *inx-18p* was PCR-amplified from *inx-18p::wCherry* and then fused to *TagBFP* amplified from *myo-3p::TagBFP* (a gift from H. Bülow) to generate *inx-18p::TagBFP* PCR fragment. *inx-18p* drives expression in the sex-shared interneuron AVG (occasionally in URX) (Oren-Suissa et al., 2016).

*eat-4p11::gfp*

*eat-4p11::gfp* was described previously (Serrano-Saiz et al., 2017a). *eat-4p11* is highly expressed in the sex-shared sensory/interneuron PHC in males but hardly detectable in hermaphrodites (Serrano-Saiz et al., 2017a).

*casy-1* constructs for rescue experiment

To generate *inx-18p::casy-1* and *eat-4pA::casy-1*, *casy-1* cDNA was PCR-amplified from *casy-1p::casy-1* (Ikeda et al., 2008) with restriction sites of KpnI and NaeI, and then digested and ligated into the KpnI/NaeI sites of *inx-18p::wCherry* and *eat-4pA::wCherry*, respectively. To generate *eat-4p11::casy-1*, the promoter region of *inx-18p::casy-1* was replaced by *eat-4p11* using restriction sites of SphI and XmaI.

To generate *inx-18p::casy-1::yfp*, *casy-1* cDNA was PCR-amplified from *casy-1p::casy-1* and then fused to *yfp* amplified from *pPD136.64* vector. This PCR fusion product was digested and ligated into the KpnI/NaeI sites of *inx-18p::wCherry* to replace *wCherry* with *casy-1::yfp*.

*casy-1* deletion constructs with *ins-1* promoter were described previously (Ikeda et al., 2008). To generate *inx-18p::mRFP::casy-1*(Full-Nt), *inx-18p::mRFP::casy-1-ΔNt* (ΔNt), *inx-18p::mRFP::casy-1-ΔCads* (ΔCads), and *inx-18p::mRFP::casy-1-ΔLNS* (ΔLNS), each of *mRFP*-tagged *casy-1* fragments was PCR-amplified from the *ins-1p* driven constructs with restriction sites of KpnI and NaeI. These PCR products were digested and ligated into the KpnI/NaeI sites of *inx-18p::wCherry* to replace *wCherry* with the *mRFP*-tagged *casy-1* fragments.

*rig-6* constructs for rescue experiment

*rig-6* cDNA that encodes a long isoform RIG-6d was obtained from total RNA of worms using iScript cDNA synthesis Kit (Bio-Rad) by two rounds of PCR reactions (primer F: 5´-actacaacgATGATGATGCTT-3´ and primer R: 5´-actgggatgaaagaattgggaac-3´ for the first round; primer F: 5´-GGGGGGTACCATGA TGATGCTTATTCGGTGTATT-3´ and primer R: 5´-GGGGTTTAAACTTAGAGTCTCCATAGTAATAATAA-3´ for the second round). The PCR product was digested with KpnI and PmeI and ligated into the KpnI/NaeI sites of *inx-18p::wCherry* to generate *inx-18p::rig-6*. The *rig-6* cDNA sequence was verified by sequencing.

To generate *eat-4pA::rig-6* and *eat-4p11::rig-6*, *eat-4pA* and *eat-4p11* fragments derived from *eat-4pA::wCherry* and *eat-4p11::gfp* with SphI/KpnI digestion were ligated into the SphI/KpnI sites of *inx-18p::rig-6* to replace *inx-18p*.

To generate *inx-18p::yfp::rig-6*, the *rig-6* signal sequence plus *yfp* was PCR-amplified from *pPD136.64* vector and fused to the remaining sequence of *rig-6* cDNA amplified from *inx-18p::rig-6* (primer F: 5´-CCCCGGTACCATGATGATGCTTATTCGGTGTATT-3´ and primer R: 5´-GGGGAGGCCTTTAGAGTCT CCATAGTAATAATAA-3´). This PCR fusion product was digested with KpnI and StuI and ligated into the KpnI/NaeI sites of *inx-18p::wCherry* to replace *wCherry* with *yfp::rig-6*.

*rig-6* deletion constructs including *inx-18p::yfp::rig-6-Δ1-3Ig* (Δ1-3Ig), *inx-18p::yfp::rig-6-Δ4-6Ig* (Δ4-6Ig), and *inx-18p::yfp::rig-6-ΔFnIII* (ΔFnIII), were generated by mutagenesis of *inx-18p::yfp::rig-6* (Full) using Q5 Site-Directed Mutagenesis Kit (New England Biolabs). The sequence was verified by sequencing. The full-length of *rig-6* cDNA is 3591 bp and deleted regions for the constructs are: 430 ~ 1320 bp for Δ1-3Ig, 1321 ~ 2199 bp for Δ4-6Ig, and 2200 ~ 3504 bp for ΔFnIII.

*bam-2* constructs for rescue experiment

*bam-2* cDNA was obtained from the *yk2037j22* clone (a gift from Y. Kohara). The sequence was verified by sequencing. To generate *eat-4pA::bam-2* and *eat-4p11:: bam-2*, a full-length *bam-2* cDNA was PCR-amplified from the *yk2037j22* clone with restriction sites of KpnI and NaeI, and then digested and ligated into the KpnI/NaeI sites of *eat-4pA::wCherry* and *eat-4p11::casy-1*, respectively.

*sax-7* constructs for rescue experiment

*ttx-3p::sax-7s* and *ttx-3p::sax-7l* were described previously (Díaz-Balzac et al., 2015). To generate *eat-4pA::sax-7s* and *eat-4pA::sax-7l*, *eat-4pA* fragment derived from *eat-4pA::casy-1* with HindIII/KpnI digestion was ligated into the HindIII/KpnI sites of *ttx-3p::sax-7s* and *ttx-3p::sax-7l* to replace *ttx-3p*. To generate *eat-4p11::sax-7s* and *eat-4p11::sax-7l*, *eat-4p11* fragment derived from *eat-4p11::gfp* with HindIII/KpnI digestion was ligated into the HindIII/KpnI sites of *ttx-3p::sax-7s* and *ttx-3p::sax-7l* to replace *ttx-3p*.

Constructs for mammalian cell expression

To generate *pCMV8::3xFLAG::casy-1*, *casy-1* cDNA was PCR-amplified without the start (ATG) and signal sequence with restriction sites of NotI and XmaI, and then digested and ligated into the NotI/XmaI sites of *pCMV8::3xFLAG::egl-15A* (Díaz-Balzac et al., 2015) to replace *egl-15A* with *casy-1*.

To generate *pCMV8::3xFLAG::rig-6*, *rig-6* cDNA was PCR-amplified without the start (ATG) and signal sequence with restriction sites of NotI and StuI, and then digested and ligated into the NotI/SmaI sites of *pCMV8::3xFLAG::egl-15A* to replace *egl-15A* with *rig-6*.

*pcDNA3.1::sax-7s::V5* was described previously (Díaz-Balzac et al., 2015). To generate *pcDNA3.1::bam-2::V5*, *bam-2* cDNA was amplified without the stop codon with restriction sites of KpnI and ApaI, and then digested and ligated into the KpnI/ApaI sites of *pcDNA3.1::sax-7s::V5* to replace *sax-7s* with *bam-2*.

To generate *pcDNA3.1::nrx-1::V5*, *nrx-1* cDNA was amplified from *gcy-8p::BirA::nrx-1* (Desbois et al., 2015) without the stop codon with restriction sites of KpnI and ApaI, and then digested and ligated into the KpnI/ApaI sites of *pcDNA3.1::sax-7s::V5* to replace *sax-7s* with *nrx-1*.

**Transgenic strains**

| Strain name | Transgene name | DNA on array | Strain background |
| --- | --- | --- | --- |
| Reporter strains | | | |
| EM1322 | *bxEx263* | *eat-4pA::gfp 25ng/μl, eat-4pA::mCherry::RAB-3 10ng/μl* | *him-5(e1490) V* |
| EM1330 | *bxIs25* | *eat-4pA::gfp 25ng/μl, eat-4pA::mCherry::RAB-3 10ng/μl* | *him-5(e1490) V* |
| EM1404 | *bxEx298* | *eat-4pA::gfp 25ng/μl, inx-18p::gfp 25ng/μl, eat-4pA::mCherry::RAB-3 25ng/μl* | *him-5(e1490) V* |
| EM1462 | *bxIs29* | *eat-4p11::gfp 50ng/μl, eat-4pA::wcherry 25ng/μl, inx-18p::TagBFP 25ng/μl* | *him-5(e1490) V* |
| TH502 | *ddIs290* | *sax-7::TY1::EGFP::3xFLAG(92C12) + unc-119(+)* | *unc-119(ed3) III* |
| OH13645 | *otIs518* | *eat-4^fosmid^::sl2::mCherry::h2b + pha-1(+)* | *pha-1(e2123) III;*  *him-5(e1490) V* |
|  | *otIs564* | *unc-47^fosmid^::sl2::mCherry::h2b + pha-1(+)* | *pha-1(e2123) III;*  *him-5(e1490) V* |
| Strains for protein localization | | | |
| EM1463 | *bxEx312* | *inx-18p::casy-1::yfp 5ng/μl, inx-18p::wCherry 25ng/μl, ttx-3::gfp 50ng/μl* | *him-5(e1490) V* |
| EM1536 | *bxEx334* | *inx-18p::yfp::rig-6 25ng/μl, inx-18p::wCherry 25ng/μl, ceh-22p::gfp 50ng/μl* | *him-5(e1490) V* |
| Strains for rescue experiments | | | |
| EM1505 | *bxEx287* | *inx-18p::casy-1 25ng/μl, ttx-3::mCherry 25ng/μl* | *casy-1(tm718) II; him-5(e1490) V* |
|  | *bxIs29* | see above |  |
| EM1506 | *bxEx285* | *eat-4pA::casy-1 25ng/μl, ttx-3::mCherry 25ng/μl* | *casy-1(tm718) II; him-5(e1490) V* |
|  | *bxIs29* | see above |  |
| EM1507 | *bxEx304* | *eat-4p11::casy-1 25ng/μl, ttx-3::gfp 50ng/μl* | *casy-1(tm718) II; him-5(e1490) V* |
|  | *bxIs29* | see above |  |
| EM1514 | *bxEx312* | see above | *casy-1(tm718) II; him-5(e1490) V* |
|  | *bxIs29* | see above |  |
| EM1513 | *bxEx289* | *inx-18p::mRFP::casy-1 5ng/μl, ceh-22::gfp 25ng/μl* | *casy-1(tm718) II; him-5(e1490) V* |
|  | *bxIs29* | see above |  |
| EM1520 | *bxEx292* | *inx-18p::mRFP::casy-1-ΔNt 5ng/μl, ceh-22::gfp 25ng/μl* | *casy-1(tm718) II; him-5(e1490) V* |
|  | *bxIs29* | see above |  |
| EM1508 | *bxEx294* | *inx-18p::mRFP::casy-1-ΔCads 5ng/μl, ceh-22::gfp 25ng/μl* | *casy-1(tm718) II; him-5(e1490) V* |
|  | *bxIs29* | see above |  |
| EM1509 | *bxEx296* | *inx-18p::mRFP::casy-1-ΔLNS 5ng/μl, ceh-22::gfp 25ng/μl* | *casy-1(tm718) II; him-5(e1490) V* |
|  | *bxIs29* | see above |  |
| EM1491 | *bxEx306* | *inx-18p::rig-6 5ng/μl, ttx-3::gfp 50ng/μl* | *rig-6(ok1589) II; him-5(e1490) V* |
|  | *bxIs29* | see above |  |
| EM1492 | *bxEx308* | *eat-4pA::rig-6 5ng/μl, ttx-3::gfp 50ng/μl* | *rig-6(ok1589) II; him-5(e1490) V* |
|  | *bxIs29* | see above |  |
| EM1493 | *bxEx310* | *eat-4p11::rig-6 5ng/μl, ttx-3::gfp 50ng/μl* | *rig-6(ok1589) II; him-5(e1490) V* |
|  | *bxIs29* | see above |  |
| EM1527 | *bxEx334* | see above | *rig-6(ok1589) II; him-5(e1490) V* |
|  | *bxIs29* | see above |  |
| EM1543 | *bxEx340* | *inx-18p::yfp::rig-6-Δ1-3Ig 5ng/μl, ttx-3::gfp 50ng/μl* | *rig-6(ok1589) II; him-5(e1490) V* |
|  | *bxIs29* | see above |  |
| EM1545 | *bxEx342* | *inx-18p::yfp::rig-6-Δ4-6Ig 5ng/μl, ttx-3::gfp 50ng/μl* | *rig-6(ok1589) II; him-5(e1490) V* |
|  | *bxIs29* | see above |  |
| EM1547 | *bxEx344* | *inx-18p::yfp::rig-6-ΔFnIII 5ng/μl, ttx-3::gfp 50ng/μl* | *rig-6(ok1589) II; him-5(e1490) V* |
|  | *bxIs29* | see above |  |
| EM1539 | *bxEx336* | *eat-4pA::bam-2 5ng/μl, ttx-3::gfp 50ng/μl* | *bam-2(cy6) I; him-5(e1490) V* |
|  | *bxIs29* | see above |  |
| EM1541 | *bxEx338* | *eat-4p11::bam-2 5ng/μl, ttx-3::gfp 50ng/μl* | *bam-2(cy6) I; him-5(e1490) V* |
|  | *bxIs29* | see above |  |
| EM1496 | *bxEx322* | *eat-4pA::sax-7l 5ng/μl, ttx-3::gfp 50ng/μl* | *sax-7(nj48) IV; him-5(e1490) V* |
|  | *bxIs29* | see above |  |
| EM1498 | *bxEx324* | *eat-4pA::sax-7s 5ng/μl, ttx-3::gfp 50ng/μl* | *sax-7(nj48) IV; him-5(e1490) V* |
|  | *bxIs29* | see above |  |
| EM1500 | *bxEx326* | *eat-4p11::sax-7l 5ng/μl, ttx-3::gfp 50ng/μl* | *sax-7(nj48) IV; him-5(e1490) V* |
|  | *bxIs29* | see above |  |
| EM1502 | *bxEx328* | *eat-4p11::sax-7s 5ng/μl, ttx-3::gfp 50ng/μl* | *sax-7(nj48) IV; him-5(e1490) V* |
|  | *bxIs29* | see above |  |
| EM1535 | *bxEx324* | see above | *him-5(e1490) V* |
|  | *bxIs29* | see above |  |
| EM1549 | *bxEx324* | see above | *casy-1(tm718) II; him-5(e1490) V* |
|  | *bxIs29* | see above |  |
| EM1550 | *bxEx324* | see above | *rig-6(ok1589) II; him-5(e1490) V* |
|  | *bxIs29* | see above |  |
| Mutant strains with reporters | | | |
| EM1414 | *bxEx298* | see above | *casy-1(tm718) II; him-5(e1490) V* |
| EM1425 | *bxEx298* | see above | *rig-6(ok1589) II; him-5(e1490) V* |
| EM1333 | *bxIs25* | see above | *oig-1(ok1687) III; him-5(e1490) V* |
| EM1334 | *bxIs25* | see above | *nlg-1(ok259) X; him-5(e1490) V* |
| EM1344 | *bxIs25* | see above | *zig-1(ok784) II; him-5(e1490) V* |
| EM1346 | *bxIs25* | see above | *casy-1(tm718) II; him-5(e1490) V* |
| EM1347 | *bxIs25* | see above | *rig-6(ok1589) II; him-5(e1490) V* |
| EM1348 | *bxIs25* | see above | *igcm-1(ok711) X; him-5(e1490) V* |
| EM1349 | *bxIs25* | see above | *oig-8(gk867223) II ;him-5(e1490) V* |
| EM1368 | *bxIs25* | see above | *nrx-1(ok1649) him-5(e1490) V* |
| EM1412 | *bxIs25* | see above | *ncam-1(hd49) X; him-5(e1490) V* |
| EM1416 | *bxIs25* | see above | *lat-1(ok1465)/mIn1 II; him-5(e1490) V* |
| EM1436 | *bxIs25* | see above | *wrk-1(ok695) X; him-5(e1490) V* |
| EM1459 | *bxIs25* | see above | *sax-7(nj48) IV; him-5(e1490) V* |
| EM1489 | *bxIs25* | see above | *nrx-1(wy778) him-5(e1490) V* |
| EM1511 | *bxIs25* | see above | *sax-3(ky123) X; him-5(e1490) V* |
| EM1531 | *bxIs25* | see above | *rig-6(gk438569) II; him-5(e1490) V* |
| EM1533 | *bxIs25* | see above | *bam-2(cy6) I; him-5(e1490) V* |
| EM1477 | *bxIs29* | see above | *rig-6(ok1589) II; him-5(e1490) V* |
| EM1481 | *bxIs29* | see above | *rig-6(ok1589) casy-1(tm718) II; him-5(e1490) V* |
| EM1482 | *bxIs29* | see above | *casy-1(tm718) II; him-5(e1490) V* |
| EM1483 | *bxIs29* | see above | *sax-7(nj48) IV; him-5(e1490) V* |
| EM1521 | *bxIs29* | see above | *rig-6(ok1589) II; sax-7(nj48) IV; him-5(e1490) V* |
| EM1522 | *bxIs29* | see above | *bam-2(cy6) I; him-5(e1490) V* |
| EM1530 | *bxIs29* | see above | *rig-6(gk438569) II; him-5(e1490)V* |
| EM1537 | *bxIs29* | see above | *bam-2(cy6) I; casy-1(tm718) II; him-5(e1490) V* |

**Strains for mating behavior**

Mutant strains were crossed at least twice into *him-5(e1490) V*. These include: *casy-1(tm718) II; him-5(e1490) V*, *rig-6(ok1589) II; him-5(e1490) V*, *bam-2(cy6) I; him-5(e1490) V*, *sax-7(nj48) IV; him-5(e1490) V*, *nlg-1(ok259) X; him-5(e1490) V*, *casy-1(tm718) rig-6(ok1589) II; him-5(e1490) V, casy-1(tm718) II; bam-2(cy6) I; him-5(e1490) V*, *rig-6(ok1589) II; sax-7(nj48) IV; him-5(e1490) V*. For rescue experiments, the following strains were used: *bxEx287; casy-1(tm718) II; him-5(e1490) V*, *bxEx306; rig-6(ok1589) II; him-5(e1490) V*, *bxEx336; bam-2(cy6) I; him-5(e1490) V*, *bxEx328; sax-7(nj48) IV; him-5(e1490) V.*

**References**

Desbois, M., Cook, S.J., Emmons, S.W., and Bülow, H.E. (2015). Directional Trans-Synaptic Labeling of Specific Neuronal Connections in Live Animals. Genetics *200*, 697–705.

Díaz-Balzac, C.A., Lázaro-Peña, M.I., Ramos-Ortiz, G.A., and Bülow, H.E. (2015). The Adhesion Molecule KAL-1/anosmin-1 Regulates Neurite Branching through a SAX-7/L1CAM-EGL-15/FGFR Receptor Complex. Cell Rep. *11*, 1377–1384.

Hobert, O. (2002). PCR fusion-based approach to create reporter gene constructs for expression analysis in transgenic *C. elegans*. Biotechniques *32*, 728–730.

Hunt-Newbury, R., Viveiros, R., Johnsen, R., Mah, A., Anastas, D., Fang, L., Halfnight, E., Lee, D., Lin, J., Lorch, A., et al. (2007). High-throughput in vivo analysis of gene expression in Caenorhabditis elegans. PLoS Biol. *5*, e237.

Ikeda, D.D., Duan, Y., Matsuki, M., Kunitomo, H., Hutter, H., Hedgecock, E.M., and Iino, Y. (2008). CASY-1, an ortholog of calsyntenins/alcadeins, is essential for learning in Caenorhabditis elegans. Proc. Natl. Acad. Sci. USA *105*, 5260–5265.
